# Serious Game for the Nursing Assessment of Home-Dwelling Older Adults: Development and Validation Study

**DOI:** 10.2196/52644

**Published:** 2024-11-26

**Authors:** Erica Busca, Silvia Caristia, Sara Palmira Bidone, Alessia Bolamperti, Sara Campagna, Arianna Cattaneo, Rosaria Lea, Doriana Montani, Antonio Scalogna, Francesca Piovesan, Erika Bassi, Alberto Dal Molin

**Affiliations:** 1 Department of Translational Medicine University of Piemonte Orientale Novara Italy; 2 Azienda Ospedaliero Universitaria Maggiore della Carità Novara Italy; 3 Department for Sustainable Development and Ecological Transition University of Piemonte Orientale Vercelli Italy; 4 Health and Socio-Health Surveillance Commission Azienda Sanitaria Locale Alessandria Alessandria Italy; 5 Interdepartmental Centre for Innovative Didactics and Simulation in Medicine and Health Professions University of Piemonte Orientale Novara Italy; 6 Department of Public Health and Pediatrics University of Torino Torino Italy; 7 University of Turin Department of Psychology BIP (BraIn Plasticity and Behaviour Changes) Research Group Turin Italy

**Keywords:** nursing education, serious game, simulation training, validation study, home-dwelling older adults, continuing education, nursing students, Family and Community Nursing, validity

## Abstract

**Background:**

The use of serious games (SGs) in nursing education is increasing, with the COVID-19 pandemic significantly accelerating their development. A key feature of SGs is their flexibility, allowing students to train at any place and time as needed. Recently, there has been a shift from developing disease-specific SGs to games focused on broader health issues. However, there has been a lack of proposals to enhance nursing interventions in home and frail care settings. The REACtion project developed a SG to improve students’ understanding and clinical reasoning in caring for home-dwelling older adults.

**Objective:**

This study aims to describe the development of “REACtion Game” (RG) and explore its validity as an educational tool. A multidisciplinary team created a SG that simulates the assessment process of older adults in home settings by nurses. It features web-based scenarios, clickable objects, and a menu with tools, and medical records to enhance nursing students’ knowledge and clinical reasoning skills.

**Methods:**

A prospective, observational study was conducted using the Dutch Society for Simulation in Healthcare’s framework to validate the game. Further, 5 experts in home health care nursing evaluated content validity, while 30 students assessed construct validity, face validity, concurrent validity (by comparing game scores with those from the Nursing Clinical Reasoning Scale), game quality, and usability. Data were collected through self-administered web-based questionnaires and the debriefings of each match played. The students were enrolled in 2 postgraduate nursing programs: a master of science in nursing degree and a first-level continuing education in family and community nursing.

**Results:**

Experts rated the content validity highly after revisions (universal agreement calculation method of scale-level content validity index=0.97). The sample consisted of 30 students, predominantly women (n=20, 67%) and aged younger than 45 years (n=23, 77%) with no prior experience in SG. Almost all students had a positive impression of RG as an attractive and useful method for learning new knowledge. Participants found the cases, scenarios, and dialogues realistic (face validity) and of high quality, though usability aspects such as instructions clarity and intelligibility of game progression were less favored. Construct validity showed general agreement on the game’s educational value, with family and community nursing students reporting more consistent alignment with educational goals. Overall, RG scores correlated positively with time spent playing but showed limited correlation with Nursing Clinical Reasoning Scale scores.

**Conclusions:**

This study developed and validated a nursing education game, especially valuable as simulation is underused in some curricula. Created during the pandemic, it offered a digital learning environment. Although the game shows potential, further testing is needed for usability, concurrent validity, and functional improvements. Future research should involve larger samples to fully validate the game and assess its impact on academic achievement.

## Introduction

In Italy, family-based and primary care–centered nursing models have recently undergone significant growth [1,2]. The rapid spread of COVID-19 highlighted the urgent need to increase primary care services to meet citizens’ increasing expectations, the aging population, and more complex health care needs [3]. The patient’s home becomes the privileged place to ensure continuity of quality care [4], where people become active participants in the care process. In this setting, nurses are required to have specific skills and advanced competencies [5], particularly in caring for frail older adults, resulting from both practical experience and graduate education [6].

In this context, the REACtion project was implemented to improve care for older adults living in little villages to preserve their functional autonomy in their life settings. A pivotal role is played by the family and community nurses, which includes health promotion and disease prevention of people in the community. An output of REACtion was the development of a serious game (SG) for the academic curricula of nurses aimed to increase their knowledge and clinical reasoning on home-dwelling older adults’ care. Clinical reasoning is a cognitive process where health care professionals gather, process, and understand patient information, plan interventions, implement them, evaluate outcomes, reflect, and learn from the experience. This process is fundamental to nursing [7].

The COVID-19 pandemic has transformed the delivery of health education, prompting the implementation of new tools for digital health education to ensure effective learning [8]. Although some studies found virtual environments to impair learning performances [9], literature shows the immersiveness of digital environments can overcome the obstacles posed by digital equipment and significantly improve engagement, providing an enhancement of learning processes and increasing motivation [10]. Simultaneously, the expanding realm of digital technology has brought heightened attention to the development of digital health education, particularly of SGs. SGs are educational games providing immersive, self-regulated training and reproducing authentic situations in a virtual environment that is safe and enjoyable [11]. SGs include features such as challenging goals, an engaging design, and scoring systems to improve player involvement during interaction and goal achievement [12]. SGs provide player immersion through fiction storylines, freedom of navigation, interactivity with objectives, and problem-solving opportunities [13]. These specific elements are thought to deeply engage players to repeatedly take on challenges to improve in-game performance and, as a result, knowledge and skills in different nursing core competencies, including management of nursing care, clinical reasoning, procedural tasks, legal practice, and quality improvement [14,15]. As SGs have been shown to be effective in higher education, they have been incorporated into the educational programs of both nursing students and nurses [16]. In recent years, there has been a gradual shift from developing disease-specific SGs toward games that focus on general health issues [17] or specific techniques. Several SGs have been developed for nursing students, aiming to enhance their knowledge in various fields such as influence vaccinations [18,19], interprofessional teamwork [20], drug preparation and administration [21], and teaching correct inhalation techniques to patients [22]. In nursing education for older adults in an extrahospital setting, the studies focused predominantly on exploring the experience of students using a SG for learning environmental hazard and safety assessment [23] or in preparation for clinical internships in home health care [24], using a qualitative approach without testing validity.

The player experience can be significantly influenced by the SG’s validity, so it is important to assess it before extensively introducing the game into education [25]. The Dutch Society for Simulation in Healthcare [26] provided the first consensus-based framework reported by Giunti et al [27] for evaluating SG applied to health care to compare and validate it consistently. Features related to game characteristics, rationale, functionality, validity, and data protection are the 5 main areas described in the framework [28]. The “classical” concepts of validity (content validity, face validity, construct validity, concurrent validity, and predictive validity) were included in the framework as they are most frequently used in validity research in medicine. To date, research addressing the development and evaluation process of SGs in the field of health education is still quite limited, although there is strong interest in their development.

Considering the importance of using validated training tools to ensure the quality and efficacy of education, this study aims to describe the validation process during the development of a SG called “REACtion Game (RG).” More specifically, this study primarily describes how the RG was developed, the herein results about the content, construct, face, and concurrent validity of the game, and results about its quality and usability. The results of the full development process of RG were herein not shown. The RG was developed as a tool for training nursing students, empowering them to perform an initial assessment of home-dwelling older adults using the gaming algorithm, thereby enhancing their clinical reasoning. This study also aims to test whether there are any potential differences in gameplay performance and clinical reasoning among participants based on (1) their course of study, (2) age, (3) work setting (specifically, primary care vs other settings), and (4) prior experience with serious and virtual games.

## Methods

### RG: The Development Process

The RG was developed by a company specializing in SG development, in collaboration with a multidisciplinary team from the University of Piemonte Orientale. Nursing experts were fully involved in the design of RG, as well as social workers, university professors with long experience in teaching, and simulation technologists with a general knowledge of simulation and scenario design principles and evaluation approaches. The multidisciplinary team was composed to design and realize the game with the complicity of the major experts on the topic and the learning tool. The RG prototype was developed from February to September 2022, with monthly meetings held during this period. The multidisciplinary team aimed to establish learner profile, and learning objectives, determine game modes, and draft dialogues for scenarios considering the context where play or learning takes place, the learner specification (age, education, and academic curricula), the mode of representation (fidelity, interactivity, and immersion levels), and pedagogic issues as learning models and approaches [29]. The RG aims to improve nursing students’ clinical reasoning and knowledge in caring for home-dwelling older adults. Specifically, it focuses on teaching how to conduct a systematic nursing assessment of older adults in a home environment and recognize active informal networks that are resources for patient care. The RG was developed concerning (1) the specific scope of practice for family and community nursing (FCN) at the national level, and (2) the characteristics of Italian older adults, who are increasingly living alone but near their children [30].

RG is a single-player game that offers an web-based experience designed for learning by doing. The player can choose 5 different scenarios reproducing real-world situations (plausible situations like a patient with chronic obstructive pulmonary disease, a lonely older adult in a mountain environment, an older adult affected by hoarding disorder, an older woman in a small group home, and an older woman with a disabled son). The selection of scenarios was discussed within the multidisciplinary team, guided by the following criteria: (1) scenarios addressable by both nursing and social work students based on their skills and (2) involving home-dwelling older adults. Developers used a validated scenario template based on learning specific objectives, resources available (ie, equipment), patient information, key actors, and critical actions [31]. The multidisciplinary team contributed their expertise to compile the contents for each scenario. Before being used by players, the RG underwent testing by technical experts and the multidisciplinary team. This verification ensured that the game operated correctly per technical aspects, including command functionality.

**Figure 1:**
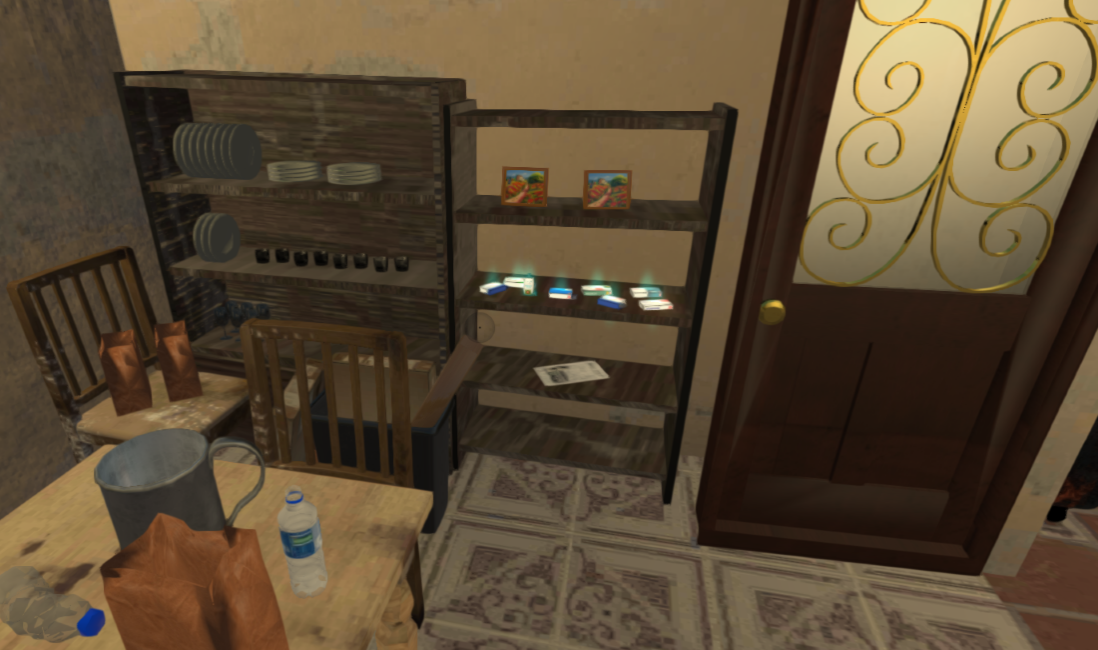
Example of clickable objects (medications) in the scenario visualized by a blue halo surrounding them. This functionality of REACtion Game allows the player to (1) access information and data or (2) unlock new actions to proceed in the game.

**Figure 2:**
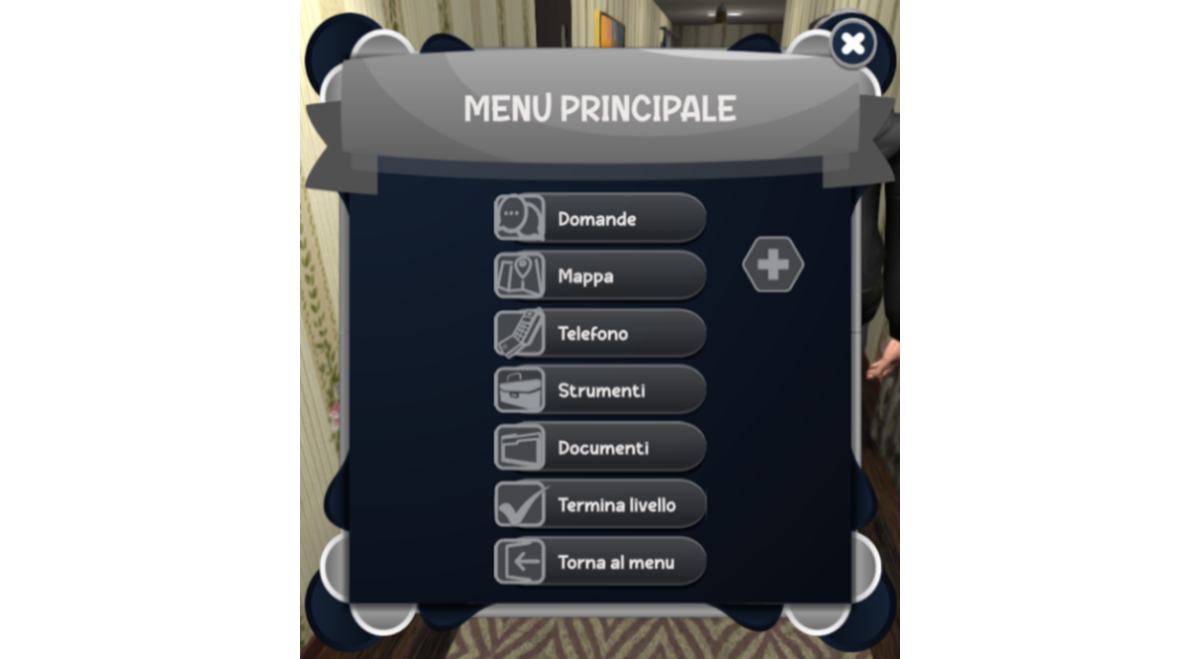
The computer’s interface of actions available from the REACtion Game menu. The game menu supplies a series of buttons useful to play and advance in the game: dialogues and questions, maps, phones, tools, and medical records.

The player, after a screen with preparatory information, can consult the clinical records, use nursing assessment tools, dialogue with the patient, interact with other actors, and explore the environment by using the mouse and keyboard commands (Figure S1 in Multimedia Appendix 1 and Figures 1 and 2). The dialogues were organized by topic, allowing players to select from a menu (ie, of topic: risk factors assessment and therapeutic adherence assessment). Players have the option to choose questions by clicking Y (yes) or N (no). Each question has only 1 correct option, and the patient’s response is automatically displayed. The scenarios’ progression is contingent on the execution of specific key actions; failure to take key actions prevents the unlocking of subsequent steps.

Each scenario can be played without any time limits. The time spent in the scenario (hours, minutes, and seconds) is shown in the debriefing. The scenario finishes when the player thinks they have completed the available actions by clicking “end level.” The player receives a score for each correct action, a final total score is provided in the debriefing, and a list of actions performed is also returned to the player. The highest possible total score is based on correct actions within each scenario. The RG was developed using the PlayCanvas engine to make it available on PCs and laptops.

### Validation of the SG

#### Design

This study’s design followed the framework of the Dutch Society for Simulation in Healthcare for the validation process [26,28], assessing the following dimensions of validity: (1) content validity is defined as “the steps taken to ensure that assessment items (including scenarios, questions, and response options) reflect the concept they are intended to measure”; (2) construct validity is defined as the grade of coherence of skills measured by the SG and the underlying theory (educative values); (3) face validity is that which answers the question “The players view it as a valid way of instruction?”; that is, face validity assesses whether the players perceive the instructional method as legitimate and effective; and (4) concurrent validity is defined as the relationships between the RG scoring and results obtained through another tool assessing the same construct.

In addition to the listed dimensions, the quality and usability of the game were also investigated.

#### Participants and Enrollment

In the validation stage, during September 2022, five experts in FCN, who had worked in a home health care setting for at least 5 years, were recruited to assess the content validity using the snowball technique; from October 2022 to December 2022 a prospective, observational study was conducted on a convenience sample of 30 volunteers’ students to examine the construct, face, concurrent validity, and game quality and usability. Inclusion criteria for students were being an undergraduate nursing student enrolled with a master of science in nursing (MSN) degree (University of Piemonte Orientale) and first-level continuing education in FCN. The latter is a 1-year program at the University of Piemonte Orientale and University of Turin that aims to develop specialized skills in community nursing. Students were enrolled voluntarily by the nursing programs’ coordinators, following an informational meeting with the researchers, who explained this study’s procedures. The researchers then collected informed consent and invited students to complete a web-based questionnaire to assess concurrent validity. Afterward, the students played the modified version of the RG, which had been updated based on the content validity feedback from 5 experts. A tutorial for students with game instructions was prepared and they had the opportunity to familiarize themselves with RG after visualizing the tutorial. Subsequently, participants recorded the score obtained for each scenario, and they were invited to answer a second web-based questionnaire investigating the other dimensions of validity.

#### Data Collection and Instrument

Data were collected by self-administered standardized questionnaires disseminated on the web through REDCap (Research Electronic Data Capture; Vanderbilt University) software (version 6.11.5). Researchers, who collected data, did not take part in the development of the game. The questionnaires were used to gather data from both experts and students who underwent preliminary testing with a small group of nurses (N=3). The questionnaire administered to experts was custom-made based on the contents of the game, whereas the questionnaire used for students was adapted from Wu et al [32], with modifications made to align the items with the RG and its context.

To evaluate content validity, 5 experts were invited to evaluate 5 factors associated with RG issues and scenarios: clinical instruments proposed by the game for play tools, the necessary actions to proceed in the game, the dialogues, the relational features between the avatar and other characters appearing in the game, and the environment. Items were graded on a 5-point Likert scale based on their importance, ranging from 1 (not important) to 5 (extremely important). Comments and suggestions were additionally required, as well as the reasons for the negative judgments.

Concurrent validity was evaluated by comparing the RG scores to the score of the Nursing Clinical Reasoning Scale (NCRS) [33]. The RG aimed to assess the skills related to clinical reasoning, considering that clinical reasoning is developed during the academic training course and the work experience as a nurse. The NCRS is a 5-point Likert scale (from 1=strongly disagree to 5=strongly agree) that measures clinical reasoning competence. The highest possible summed score for NCRS is 75. The Cronbach α coefficient of 0.90 showed good internal consistency [33]. Immediately before playing RG, students filled in a web-based questionnaire including the NCRS to avoid any game-related contamination. Participants were successively invited to download the first debriefing of each scenario played and upload their scores to the didactic platform. Data on RG and NCRS scoring were gathered.

After completing the RG, students were asked to fill in a second and final web-based standardized questionnaire, which included their impressions and attitudes toward RG’s educational values (construct validity) and game quality and usability. For construct validity, the questionnaire included (12 items rated on a 5-point Likert scale to assess educational values (acquisition of knowledge, clinical and organizational skills, effectiveness in education, necessity for learning, effective feedback, sense of immersiveness, fun, willingness to play again, and long-lasting learning). Face validity was evaluated using a 5-point frequency scale (from 1=strongly disagree to 5=strongly agree) comprising 11 items regarding the realism of cases (5 items), scenarios (3 items), and dialogues (3 items). Items were created by adapting items used by Wu et al [32]. The quality and usability of the game were assessed using a 5-point Likert scale (from 1=strongly disagree to 5=strongly agree) to collect opinions on the quality of sound, images (1 item), the sensations recall by each scenario (3 items), the goodness of the game (1 item), and the game’s ease of use for a novice player (4 items). The Cronbach α coefficients of 0.75 for face validity, quality, and usability of the game and 0.93 for educational values (construct validity) showed good internal consistency.

Finally, sociodemographic data, nursing experience in primary care, serious and virtual game experience, and the number of matches played were collected through questionnaires administered to the student sample. In addition, the score for each scenario and the length of each match from the debriefing were recorded.

### Analysis

Content validity: first, for each item, the item-level content validity index (I-CVI) was calculated as the proportion of “relevant” judgments (number of experts who rated the item as either 4 or 5, “relevant” and “highly relevant,” respectively) on the total number of experts. Considering the small sample (5 experts), only items with an I-CVI=1 were retained; by contrast, I-CVI<1 items were modified or dropped. Second, the scale-level content validity index was calculated using the universal agreement calculation methods (S-CVI/UA): number of items with I-CVI=1 on the total number of items. The full “scale” is valid with a scale-level content validity index ≥0.80 (80% of agreement among experts) [34].

Descriptive analyses were carried out for RG and NCRS scores and 1-way ANOVA was used to test differences between courses. To evaluate concurrent validity, RG scores were correlated to NCRS scores using the Spearman correlation for the nonlinear nature of the relation between the 2 variables. For all statistical tests, a significance level of *P*<.05 was used.

All Likert scales and partial semantic autonomy scales used to measure face validity, usability, and quality of RG, were changed in dichotomous variables (agree vs disagree or uncertain position) and prevalence was reported by courses. Based on the type of variable, the Fisher exact test or Student *t* test (2-tailed) was used to test differences with a significant level of *P*<.05.

To address potential confounding effects, factors such as age, previous experience with serious and virtual games, gender, work setting, and course membership were incorporated into the analyses. A limited time window for game use was implemented to maintain concurrent validity. Additionally, anonymity and self-completion of the questionnaire aimed to reduce the likelihood of social desirability bias.

### Ethical Considerations

This study was approved by the Interagency Ethics Committee of Novara (protocol 821/CE). Written informed consent was obtained from all participants. Data on match play were not obtained from the RG repository. Instead, players downloaded the data after each match and provided it to researchers via the university’s didactic e-platform in a digital storage area accessible only to the researchers. The data were pseudonymized: each nursing student involved in this study was associated with a unique identifier given by the order of completion of the web-based questionnaires. No remuneration was provided for participation in this study.

## Results

### Content Validity

The original S-CVI/UA ranged from 0.25 to 1.00, with 9 items below 0.75 related to dialogue between characters (nurse and patient, family members, and other professionals). After revision, the total S-CVI/UA increased from 0.95 to 0.97 (Table 1).

**Table 1:** The scale-level content validity index of REACtion Game themes.

Themes	Items, n	S-CVI/UA^a^	
Environment	10	1.00	N/A^b^
Materials and tools	18	1.00	N/A
Activities	30	1.00	N/A
Dialogues	157	0.92	0.96
Relationships	6	1.00	N/A
Total	221	0.95	0.97

^a^S-CVI/UA: number of items with item-level content validity index=1 on the total number of items.

^b^N/A: not applicable.

### Sample Characteristics

Table 2 shows the main characteristics of the sample (N=30). Among students enrolled, the response rate was 100% in both questionnaires. Women (n=20, 67%) and younger students (n=23, 77% were younger than 45 years) made up a considerable proportion of the sample. More than two-thirds of the participants were from the FCN group (n=21). Further, 10 students worked in a primary care setting (33%), while 11 (37%) were employed in a hospital. Nobody declared any prior experience with serious and virtual games. Only 5 (17%) participants played 1 match for each scenario, with an average total playing time of 86 (SD 37.8) minutes. Comparison between course groups did not show significant differences (Table 1) for gender, age, and working experiences. The mean time spent playing is significantly higher in the FCN group.

**Table 2:** Characteristics of the student sample and data on game played by participants in the 2 post graduate programs.

			Total (N=30)		MSN^a^ (n=9)		FCN^b^ (n=21)		*P* value^c^
**Gender^d^, n (%)**									
	Women	20 (67)		7 (78)		13 (62)		.68	
**Age (years), n (%)**									
	≤44	23 (77)		8 (89)		15 (71)		.39	
	45-65	7 (23)		1 (11)		6 (29)		N/A^e^	
**Workplace setting, n (%)**									
	Primary care	10 (33)		8 (89)		12 (57)		.09	
	Other	20 (67)		1 (11)		9 (43)		N/A	
**Worked in primary care setting (years), n (%)**									
	Less than 2	7 (70)		1 (100)		6 (67)		≥.99	
	More than 2	3 (30)		N/A		3 (33)		N/A	
**Matches played for each scenario, n (%)**									
	Only 1 match	5 (17)		3 (33)		2 (10)		.14	
	More than 1	25 (83)		6 (67)		19 (91)		N/A	
**Time spent for each match (min), mean (SD)**									
	Scenario no 1	22.7 (31)		13.8 (2)		26.5 (8)		.31	
	Scenario no 2	19.8 (10)		14 (2)		22.2 (2)		.04	
	Scenario no 3	18.5 (11)		13.3 (3)		20.9 (3)		.08	
	Scenario no 4	18.3 (10)		13.1 (2)		20.5 (2)		.05	
	Scenario no 5	17 (21)		10.3 (2)		19.8 (5)		.25	
	All scenarios	85.9 (37)		64.7 (9)		95 (8)		.04	

^a^MSN: master of science in nursing.

^b^FCN: family and community nursing.

^c^Fisher exact test or Student *t* test.

^d^To detect gender information, we asked participants to choose among these 3 gender identity options: (1) woman, (2) man, and (3) nonbinary.

^e^N/A: not applicable.

### Face, Quality, and Usability of RG

Table 3 shows the prevalence of participants who agree with items on face validity, quality, and usability of RG. Almost all participants thought the cases, scenarios, and dialogues were realistic. The percentages of agreement were high for game quality but lower for aspects of usability (intelligibility of instructions, command, and game progress). There were no significant differences between the participants in the 2 groups for any item (Table 3) as well as between age classes, workplace settings (primary care vs others), and number of matches played (Multimedia Appendix 1). A significant difference was only found between gender in the intelligibility of the game process (item 11; Multimedia Appendix 1).

**Table 3:** Prevalence of agreement (sum of “agree” and “strongly agree” responses) on the domains of face validity, quality, and usability of REACtion Game by post graduate program.

			Prevalence of agreement						
			MSN^a^ (n=9), n (%)		FCN^b^ (n=21), n (%)		Total (N=30), n (%)		*P* value^c^
**Domains for face validity (16 items)**									
	Verisimilitude of cases (5 items)	8 (89)		19 (91)		27 (90)		.99	
	Verisimilitude of scenarios (3 items)	8 (89)		19 (91)		27 (90)		.99	
	Verisimilitude of dialogues with patients	9 (100)		20 (95)		29 (97)		.99	
	Verisimilitude of dialogues with family members	8 (89)		19 (91)		27 (90)		.99	
	Verisimilitude of dialogues with other professionals	7 (78)		14 (67)		21 (70)		.68	
**Domains for quality and usability of the game**									
	Sensation recalled by scenario (overall; 3 items)	9 (100)		21 (100)		30 (100)		N/A^d^	
	Goodness of the game	8 (89)		20 (95)		28 (93)		.52	
	Quality of image and sound	7 (78)		15 (71)		22 (73)		.99	
	Intelligibility of instructions	6 (67)		14 (67)		20 (67)		.99	
	Intelligibility of command use	5 (56)		10 (48)		15 (50)		.99	
	Intelligibility of the game progress	5 (56)		10 (48)		15 (50)		.99	
	Debriefing usefulness	6 (67)		10 (48)		16 (53)		.44	

^a^MSN: master of science in nursing.

^b^FCN: family and community nursing.

^c^Fisher exact test.

^d^N/A: not applicable.

### Construct Validity

Table 4 shows the prevalence of respondents who agreed with the 12 items used to evaluate construct validity. In total, 13 (62%) students in the FCN group, compared to 5 (55%) of those in the MSN group, declared that RG was consistent with the educational values. Students reported that the most positive impression of RG was “acquisition of information useful for understanding the single situation” followed by “acquisition of skills to identify priority and goals” and “the effective feedback,” with a prevalence of over 70%. Further, 4 items received slight agreement (prevalence around 40%; Table 4). No significant differences were found between the 2 groups, as well as between gender, age classes, workplace settings (primary care vs others), and number of matches played (Multimedia Appendix 1).

**Table 4:** Prevalence of agreement (sum of “agree” and “strongly agree” responses) on the items of the construct validity of the game by post graduate program.

Items for construct validity	MSN^a^, (n=9), n (%)	FCN^b^, (n=21), n (%)	Total (N=30), n (%)	*P* value^c^
New knowledge acquisition	5 (56)	14 (67)	19 (63)	.69
Acquisition of information useful for understanding the single situation	6 (67)	20 (95)	26 (87)	.06
Professional development	4 (44)	13 (62)	17 (57)	.44
Acquisition of skills to identify priorities and goals	7 (78)	14 (67)	21 (70)	.68
Development of organizational skills	6 (67)	14 (67)	20 (67)	.99
Development of clinical skills	2 (22)	10 (48)	12 (40)	.25
Effective feedback	5 (56)	16 (76)	21 (70)	.26
The game is captivating	5 (56)	15 (71)	20 (67)	.39
Play is pleasant	4 (44)	11 (52)	15 (50)	.43
Is it pleasant to play again?	4 (44)	8 (38)	12 (40)	.99
The game transfers long-term knowledge	3 (33)	11 (52)	14 (47)	.44
The training experience is essential for learning	3 (33)	10 (48)	13 (43)	.69

^a^MSN: master of science in nursing.

^b^FCN: family and community nursing.

^c^Fisher exact test.

### Concurrent Validity

Table 5 shows the mean scores for both the NCRS scale and RG scenarios. The highest possible summed score for NCRS was 75, and results showed a rather high mean overall score (58, SD 6.1). The scores from the MSN group (62.1, SD 3.9) were significantly higher than the scores from the FCN group (56.8, SD 6.2, *P*=.03). Total mean RG scores for MSN and FCN students were 101.3 (SD 33. 6) and 154.8 (SD 36), respectively, and the difference between groups was statistically significant (*P*=.001). Although the overall RG score was slightly higher for the FCN group, there were no statistically significant group differences noted for scenario number 3. Further analysis revealed that no significant differences in NCRS and RG scores were found when considering the gender and the age classes except for RG scores in scenario number 5 (Multimedia Appendix 1). According to the results, the mean NCRS score was lower in students working in a primary care setting compared to students employed in other workplace settings (55.8, SD 6.8 vs 60.1, SD 5, *P*=.05; Multimedia Appendix 1). Additionally, a significant positive correlation emerged between RG scores and the time spent playing (Pearson coefficient 0.604, *P*<.001). Finally, we did not find any correlation between NCRS scores and RG total scores, except for scenario number 1 played by the MSN group (Spearman coefficient 0.73, *P*=.03; Table 6) and for scenario number 2 where students played only 1 match (Multimedia Appendix 1).

**Table 5:** NCRS^a^ scale scores and REACtion Game scores for each scenario by post graduate program.

Score	Total (N=30), mean (SD)	MSN^b^ (n=9), mean (SD)	FCN^c^ (n=21), mean (SD)	*P* value^d^
NCRS scale^e^	58.40 (6.08)	62.11 (3.92)	56.81 (6.21)	.03^f^
RG^g^ scenario no^h^ 1	31.78 (19.39)	15.22 (12.24)	38.88 (17.57)	.001^f^
RG scenario no 2	34.67 (11.49)	26.94 (11.52)	37.98 (10)	.01^f^
RG scenario no 3	28.48 (8.90)	25.72 (10.07)	29.67 (8.33)	.27
RG scenario no 4	29.90 (8.31)	25.47 (10.89)	31.8 (6.32)	.05^f^
RG scenario no 5	13.93 (7.1)	7.94 (7.3)	16.5 (5.35)	.001^f^
RG all scenarios	138.77 (42.77)	101.3 (33.56)	154.82 (36.04)	.001^f^

^a^NCRS: Nursing Clinical Reasoning Scale.

^b^MSN: master of science in nursing.

^c^FCN: family and community nursing.

^d^ANOVA test.

^e^NCRS scale: Scores range from 0 to 75, higher scores mean higher clinical reasoning skills. Scenario number 1: scores range from 0 to 58. Scenario number 2: scores range from 0 to 51. Scenario number 3: scores range from 0 to 38. Scenario number 4: scores range from 0 to 41. Scenario number 5: scores range from 0 to 20. All scenarios: scores range from 0 to 208. Higher game scores mean better play performance.

^f^*P* values below .05.

^g^RG: REACtion Game.

^h^no: number.

**Table 6:** Correlation between NCRS^a^ scale scores and REACtion Game scores, by scenario and postgraduate program.

RG^b^ score	Total (N=30)			MSN^c^ (N=9)			FCN^d^ (N=21)	
	ρ^e^	*P* value	ρ^e^		*P* value	ρ^e^		*P* value
Scenario no^f^ 1	–0.032	.87	0.729		.03	0.202		.38
Scenario no 2	0.018	.92	0.485		.18	0.224		.33
Scenario no 3	–0.027	.89	0.602		.09	–0.117		.61
Scenario no 4	–0.063	.74	0.359		.34	–0.082		.73
Scenario no 5	–0.118	.53	0.022		.96	0.212		.36
All scenarios	–0.034	.86	0.639		.06	0.212		.36

^a^NCRS: Nursing Clinical Reasoning Scale.

^b^RG: REACtion Game.

^c^MSN: master of science in nursing.

^d^FCN: family and community nursing.

^e^Spearman rank-order correlation.

^f^no: number.

## Discussion

### Principal Findings

This study describes the validity of a SG as an innovative teaching tool to prepare students before gaining practical experience. Although validation studies are increasing, literature provides various examples of evaluating the efficacy of SGs in nursing education [15,16] and very little evidence about SG validity used for training. For example, a recent publication showed the literature gaps in this field underlining the lack of evidence about the usability of these educational tools in undergraduate nursing education [14]. So, many SGs used in educational fields do not yet undergo validation, as this is a time-consuming and costly enterprise [35]. When choosing a SG as an educational tool, its validity is an important factor to consider [28]. In this study, we present the process of RG development and results on 5 domains of validity: content validity, construct validity, face validity, game quality, usability, and concurrent validity. All domains were observed collecting data from 5 experts and 30 nursing students using web-based self-reported questionnaires.

The content validity was demonstrated, as the experts positively assessed the game’s content and determined its legitimacy. From construct validity, results showed a higher positive impression of RG as an attractive and useful method to learn new knowledge, obtain information to help them understand the situation, and set priorities and goals. RG integrates the information acquired through the assessment of the older adult into actions that the player has to perform to continue in the game; these actions are similar to those carried out by a nurse in a home environment. Although most students perceive an immediate acquisition of knowledge, that is not the same as remembering it for a long time. Blakely et al [36] showed inconsistent results on the long-term retention of information through educational gaming. These results may have been influenced by the quick feeling of “knowing more” about the topic, which appears to be characteristic of the postgame.

Participants, in this study, evaluated the feedback as effective. RG integrates the feedback by giving the player a score once the match ends. In addition, to overcoming the limitations described for other SGs [32], information such as how long a student was logged in and what actions were taken or avoided throughout the match can be collected from the game’s logging system. Although feedback is considered a key factor in improving learning, there is no recommendation on the most effective way to integrate it in a SG [37]. In our study, 12 (40% of the total sample) participants mentioned that they wanted to play again, indicating that many participants did not find games enjoyable or helpful as reported in the literature [36]. For these students, SG represents one learning opportunity among many others. This may explain why SGs were not more motivating than conventional methods [38]. Although in our sample only 13 (43%) participants agreed that RG is helpful for learning, the game showed great potential to support clinical training when the real patient is not available. This was especially true during the COVID-19 outbreak in Italy when internship learning opportunities were limited [39,40].

Face validity, game quality, and usability were also assessed in this study. A great consensus among participants was found for the realism of cases, scenarios, and dialogues. Only 70% (n=21) of participants agreed that dialogues with other professionals were realistic. The fact that professional relationships vary depending on the work environment and are closely related to the particular context can help to explain this. Regarding the quality and usability of the RG, participants evaluated the use of the controls and the progression of the game as poorly intuitive, although a user guide was provided. Possible explanations are that the participants had never played a virtual game before and that the game could have included different kinds of support related to the selection of significant data (feedback, modeling, and modality) [37]. In fact, the game only used feedback as a tool to let the players know whether the information and actions were relevant to achieving the objectives of the RG.

Finally, simulation strategies such as SGs were used to teach clinical reasoning [41]. Concurrent validity shows no correlation between RG and NCRS scores. NCRS scores were higher among the MSN group and in nurses working in nonprimary care settings (hospital, clinical, and residential settings), whereas RG scores were higher in the FCN group. Although we cannot completely exclude the possibility that clinical reasoning is not necessary for the RG performance, we found at least two different reasons for concurrent validation failure. First, evaluating clinical reasoning learning is complex [42], and self-assessment by the NCRS only provides a subjective student’s perception of clinical reasoning competence. Second, while the NCRS has been validated for clinical situations [43], particularly in hospital settings, it is likely that some modifications of the scale are required before it can be used in the community or home health care nursing. However, correlation coefficients were positive considering separately the 2 courses, especially for the MSN group.

This study has some limitations. First, the aforementioned question related to the tool used for the concurrent validity. Second, the estimation of the minimum number of students required to validate the game was not performed; the sample was not randomized and based on volunteers, so results can be biased and the small sample could have influenced the nonsignificance of concurrent validity results. Although the results are not generalizable, we recruited students who were attending postgraduate training. It would be useful to be able to validate the game for undergraduate nurses as well as to increase the strength of evidence in support of RG validity. Finally, although the students’ items of the questionnaire were adapted by Wu et al [32], we used nonvalidated questionnaires (for experts and students) for test validity, except for the items used to measure construct validity and the NCRS for concurrent validity. We tested questionnaires with a small group of nurses to ensure that the items were clear, concise, unambiguous, and exhaustive.

While other SGs have been developed in the field of home health care, RG is the first game created for Italian nursing education. It considers the unique aspects of the nursing role in the community and home environment and the specific characteristics of older adults, including the support networks within local communities. The game’s validity was demonstrated for all domains except concurrent validity, although wider observation (increasing the size sample and including students from other universities and courses) is needed to increase the internal and external validity of results. As a result, although this version of the game cannot be used to assess student learning, it was well received by participants and included in 2 post–basic training programs.

In conclusion, this study aimed to develop and validate a game that could be used in nursing education. The game represented a significant opportunity for both the project and the academic courses, particularly in fields where simulation has not yet been fully incorporated into the academic curriculum. Developed during the pandemic, it provided students with the opportunity to immerse themselves in a computer-based learning environment. Although there is a need, for example, for further testing of the usability of the RG, concurrent validity, and improvement in some functional aspects, this study was the first step to support the use of the game in nursing education. Despite this study’s limitations, it is important to recognize the potential for growth of RG. While the findings are not robust enough to fully validate RG as a tool, they certainly point toward exciting improvement possibilities. RG has the potential to be expanded to give students a safe practice environment that simulates real-world conditions. This is especially true when the patient’s home is the learning environment, which is not typically offered as an internship in nursing education. However, future studies should include a larger sample to test the validity of the game, identify a better-validated tool for concurrent validity, and evaluate its predictive validity concerning academic achievement.

## Appendix

Multimedia Appendix 1 Tables showing the face, quality and usability of the game; construct validity; scores of NCRS scale and game scores; and correlation between NCRS scale and the game scores; with a figure showing REACtion Game scenarios. NCRS: Nursing Clinical Reasoning Scale.

## Abbreviations

FCN: family and community nursing
I-CVI: item-level content validity index
MSN: master of science in nursing
NCRS: Nursing Clinical Reasoning Scale
REDCap: Research Electronic Data Capture
RG: REACtion Game
S-CVI/UA: universal agreement calculation method of scale-level content validity index
SG: serious game

## Data Availability

The datasets generated during or analyzed during this study are available from the corresponding author upon reasonable request.
