# Correction: Serious Game for the Nursing Assessment of Home-Dwelling Older Adults: Development and Validation Study

**DOI:** 10.2196/81884

**Published:** 2025-12-17

**Authors:** Erica Busca, Silvia Caristia, Sara Palmira Bidone, Alessia Bolamperti, Sara Campagna, Arianna Cattaneo, Rosaria Lea, Doriana Montani, Antonio Scalogna, Francesca Piovesan, Erika Bassi, Alberto Dal Molin

**Affiliations:** 1 Department of Translational Medicine University of Piemonte Orientale Novara Italy; 2 Azienda Ospedaliero Universitaria Maggiore della Carità Novara Italy; 3 Department for Sustainable Development and Ecological Transition University of Piemonte Orientale Vercelli Italy; 4 Health and Socio-Health Surveillance Commission Azienda Sanitaria Locale Alessandria Alessandria Italy; 5 Interdepartmental Centre for Innovative Didactics and Simulation in Medicine and Health Professions University of Piemonte Orientale Novara Italy; 6 Department of Public Health and Pediatrics University of Torino Torino Italy; 7 BIP (BraIn Plasticity and Behaviour Changes) Research Group Department of Psychology University of Turin Turin Italy

In “Serious Game for the Nursing Assessment of Home-Dwelling Older Adults: Development and Validation Study” [1] the authors made one addition.

Francesca Piovesan has been added as a co-author on the manuscript. FR made a substantial intellectual contribution to the manuscript, specifically during the writing and critical revision phases. Her involvement was acknowledged and agreed upon by the research team, and was inadvertently omitted.

The authorship list has been changed as follows:

Erica Busca, PhD; Silvia Caristia, PhD; Sara Palmira Bidone, MSc; Alessia Bolamperti, MSc; Sara Campagna, PhD; Arianna Cattaneo, MSc; Rosaria Lea, MSc; Doriana Montani, MSc; Antonio Scalogna, MSc; Francesca Piovesan, PhD; Erika Bassi, PhD; Alberto Dal Molin, PhD

And the following affiliation has been added, with subsequent affiliations renumbered accordingly:

BIP (BraIn Plasticity and Behaviour Changes) Research Group, Department of Psychology, University of Turin, Turin, Italy

The correction will appear in the online version of the paper on the JMIR Publications website, together with the publication of this correction notice. Because this was made after submission to PubMed, PubMed Central, and other full-text repositories, the corrected article has also been resubmitted to those repositories.
