# Supplementary material for: Serious Game for the Nursing Assessment of Home-Dwelling Older Adults: Development and Validation Study
Source: JMIR Serious Games. 2024 Nov 26;12:e52644. doi: 10.2196/52644 (PMC11632285; doi:10.2196/52644)
Supplement: Multimedia Appendix 1 [file games_v12i1e52644_app1.docx]

**Table S1**. Face, quality, and usability of the game: prevalence of agreement by gender, age, workplace setting, and number of matches played.

|  | Prevalence of agreement | | | | | | | | | | | |
| --- | --- | --- | --- | --- | --- | --- | --- | --- | --- | --- | --- | --- |
|  | **Gender** | | | **Age classes** | | | **Nurse types** | | | **Number of matches by scenario** | | |
|  | **Women (N=20)** | **Men (N=10)** | **p-value*** | **<44 years (N=23)** | **≥45 years (N=7)** | **p-value*** | **Primary care nurses (N=10)** | **Other nurses (N=20)** | **p-value*** | **One (N=5)** | **More than one (N=25)** | **p-value*** |
|  | **N (%)** | **N (%)** |  | **N (%)** | **N (%)** |  | **N (%)** | **N (%)** |  | **N (%)** | **N (%)** |  |
| Verisimilitude of cases | 18 (90.0) | 9 (90.0) | 1.000 | 22 (95.6) | 5 (71.4) | 0.128 | 9 (90.0) | 18 (90.0) | 1.000 | 5 (100.0) | 22 (88.0) | 1.000 |
| Verisimilitude of scenario | 18 (90.0) | 9 (90.0) | 1.000 | 21 (91.3) | 6 (85.7) | 1.000 | 10 (100.0) | 17 (85.0) | 0.532 | 5 (100.0) | 22 (88.0) | 1.000 |
| Verisimilitude of dialogues with patients | 20 (100.0) | 9 (90.0) | 0.333 | 23 (100.0) | 6 (85.7) | 0.233 | 9 (90.0) | 20 (100.0) | 0.333 | 5 (100.0) | 24 (96.0) | 1.000 |
| Verisimilitude of dialogues with families | 17 (85.0) | 10 (100.0) | 0.532 | 20 (87.0) | 7 (100.0) | 1.000 | 9 (90.0) | 18 (90.0) | 1.000 | 4 (80.0) | 23 (92.0) | 0.433 |
| Verisimilitude of dialogues with other professionals | 15 (75.0) | 6 (60.0) | 0.431 | 17 (73.9) | 4 (57.1) | 0.640 | 7 (70.0) | 14 (70.0) | 1.000 | 5 (100.0) | 16 (64.0) | 0.286 |
| Sensation recalled by scenario | 20 (100.0) | 10 (100.0) | - | 23 (100.0) | 7 (100.0) | - | 10 (100.0) | 20 (100.0) | - | 5 (100.0) | 25 (100.0) | - |
| Goodness of the game | 18 (90.0) | 10 (100.0) | 0.540 | 22 (95.6) | 6 (85.7) | 0.418 | 10 (100.0) | 18 (90.0) | 0.540 | 5 (100.0) | 23 (92.0) | 1.000 |
| Quality of image and sound | 15 (75.0) | 7 (70.0) | 1.000 | 15 (65.2) | 7 (100.0) | 0.143 | 9 (90.0) | 13 (65.0) | 0.210 | 5 (100.0) | 17 (68.0) | 0.287 |
| Intelligibility of instructions | 14 (70.0) | 6 (60.0) | 0.690 | 15 (65.2) | 5 (71.4) | 1.000 | 8 (80.0) | 12 (60.0) | 0.419 | 3 (60.0) | 17 (68.0) | 1.000 |
| Intelligibility of command use | 10 (50.0) | 5 (50.0) | 1.000 | 12 (52.2) | 3 (42.9) | 1.000 | 6 (60.0) | 9 (45.0) | 0.700 | 4 (80.0) | 11 (44.0) | 0.330 |
| Intelligibility of the game progress | 13 (65.0) | 2 (20.0) | **0.050** | 11 (47.8) | 4 (57.1) | 1.000 | 4 (40.0) | 11 (55.0) | 0.700 | 3 (60.0) | 12 (48.0) | 1.000 |
| Debriefing usefulness | 9 (45.0) | 7 (70.0) | 0.260 | 11 (47.8) | 5 (71.4) | 0.399 | 5 (50.0) | 11 (55.0) | 1.000 | 4 (80.0) | 12 (48.0) | 0.336 |

* Fisher’s exact test

**Figure S1.** REACtion Game scenarios: external and household environments characterizing life contexts of the game cases.

[Artwork note: single figure; color reproduction only online version]


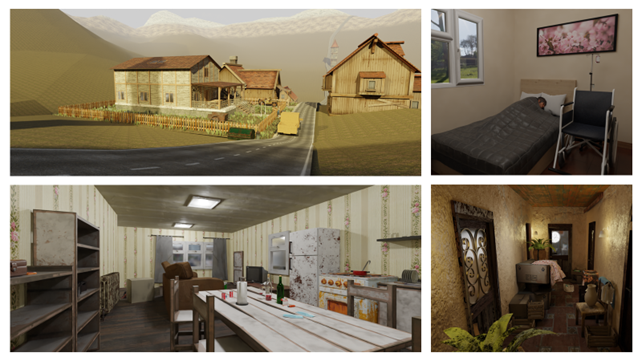


**Table S2.** Construct validity: prevalence of agreement by age, gender, workplace setting, and number of matches played.

|  | Prevalence of agreement | | | | | | | | | | | |
| --- | --- | --- | --- | --- | --- | --- | --- | --- | --- | --- | --- | --- |
|  | **Age classes** | | | **Gender** | | | **Nurse types** | | | **Number of matches by scenario** | | |
|  | **<44 years (N=23)** | **≥45 years (N=7)** | **p-value*** | **Women (N=20)** | **Men (N=10)** | **p-value*** | **Primary care nurses (N=10)** | **Other nurses (N=20)** | **p-value*** | **One (N=5)** | **More than one (N=25)** | **p-value*** |
|  | **N (%)** | **N (%)** |  | **N (%)** | **N (%)** |  | **N (%)** | **N (%)** |  | **N (%)** | **N (%)** |  |
| New knowledge acquisition | 14 (60.9) | 5 (71.4) | 1.000 | 14 (70.0) | 5 (50.0) | 0.425 | 7 (70.0) | 12 (60.0) | 0.702 | 3 (60.0) | 16 (64.0) | 1.000 |
| Acquisition of information useful for understanding the single situation | 19 (82.6) | 7 (100.0) | 0.548 | 18 (90.0) | 8 (80.0) | 0.584 | 9 (90.0) | 17 (85.0) | 1.000 | 3 (60.0) | 23 (92.0) | 0.119 |
| Professional development | 13 (56.5) | 4 (57.1) | 1.000 | 14 (70.0) | 3 (30.0) | 0.056 | 6 (60.0) | 11 (55.0) | 1.000 | 2 (40.0) | 15 (60.0) | 0.628 |
| Acquisition of ability to identify priority and goals | 15 (65.2) | 6 (85.7) | 0.393 | 15 (75.0) | 6 (60.0) | 0.431 | 8 (80.0) | 13 (65.0) | 0.675 | 3 (60.0) | 18 (72.0) | 0.622 |
| Development of organisational skills | 15 (65.2) | 5 (71.4) | 1.000 | 15 (75.0) | 5 (50.0) | 0.231 | 8 (80.0) | 12 (60.0) | 0.419 | 3 (60.0) | 17 (68.0) | 1.000 |
| Development of clinical skills | 7 (30.4) | 5 (71.4) | 0.084 | 9 (45.00) | 3 (30.0) | 0.694 | 4 (40.0) | 8 (40.0) | 1.000 | 2 (40.0) | 10 (40.0) | 1.000 |
| The feedback is effective for learning | 16 (69.6) | 5 (71.4) | 1.000 | 13 (65.0) | 8 (80.0) | 0.675 | 8 (80.0) | 13 (65.0) | 0.675 | 4 (80.0) | 17 (68.0) | 1.000 |
| The game is captivating | 14 (60.9) | 6 (85.7) | 0.372 | 13 (65.0) | 7 (70.0) | 1.000 | 9 (90.0) | 11 (55.0) | 0.101 | 3 (60.0) | 17 (68.0) | 1.000 |
| Play is pleasant | 11 (47.8) | 4 (57.1) | 1.000 | 10 (50.0) | 5 (50.0) | 1.000 | 6 (60.0) | 9 (45.0) | 0.700 | 2 (40.0) | 13 (52.0) | 1.000 |
| Is pleasant play again | 7 (30.4) | 5 (71.4) | 0.084 | 9 (45.00) | 3 (30.0) | 0.694 | 6 (60.0) | 6 (30.0) | 0.139 | 1 (20.0) | 11 (44.0) | 0.622 |
| The game transfers long-term knowledge | 10 (43.5) | 4 (57.1) | 0.675 | 10 (50.0) | 4 (40.0) | 0.709 | 5 (50.0) | 9 (45.0) | 1.000 | 2 (40.0) | 12 (48.0) | 1.000 |
| The training experience is essential for learning | 9 (39.1) | 4 (57.1) | 0.666 | 10 (50.0) | 3 (30.0) | 0.440 | 5 (50.0) | 8 (40.0) | 0.705 | 2 (40.0) | 11 (44.0) | 1.000 |

* Fisher’s exact test

**Table S3.** Scores of NCRS scale and game scores by age, gender, workplace setting, and number of matches played: descriptive results.

|  | Age classes | | | | | Gender | | | | | Nurse types | | | | | Number of matches by scenario | | | |  |
| --- | --- | --- | --- | --- | --- | --- | --- | --- | --- | --- | --- | --- | --- | --- | --- | --- | --- | --- | --- | --- |
|  | <44 years (N=23) | | ≥45 years (N=7) | |  | Women (N=20) | | Men (N=10) | |  | Primary care nurses (N=10) | | Other nurses (N=20) | |  | One (N=5) | | More than one (N=25) | |  |
|  | Mean | ±SD | Mean | ±SD | p-value* | Mean | ±SD | Mean | ±SD | p-value* | Mean | ±SD | Mean | ±SD | p-value* | Mean | ±SD | Mean | ±SD | p-value* |
| NCRS scale | 59,39 | 5,77 | 55,14 | 6,36 | 0,106 | 59,35 | 6,86 | 56,50 | 3,69 | 0,232 | 55,83 | 6,83 | 60,11 | 5,00 | **0,050** | 41,70 | 6,46 | 58,12 | 6,10 | 0,582 |
| RG score, scenario no. 1 | 32,91 | 11,69 | 40,43 | 9,34 | 0,132 | 34,85 | 10,42 | 34,30 | 14,02 | 0,904 | 33,79 | 12,94 | 35,25 | 10,78 | 0,740 | 25,90 | 22,93 | 32,96 | 18,92 | 0,467 |
| RG score, scenario no. 2 | 28,66 | 8,77 | 33,96 | 5,11 | 0,143 | 30,02 | 8,50 | 29,66 | 8,35 | 0,913 | 29,10 | 7,54 | 30,43 | 8,95 | 0,674 | 32,70 | 11,73 | 35,06 | 11,65 | 0,683 |
| RG score, scenario no. 3 | 31,28 | 19,75 | 33,43 | 19,55 | 0,803 | 34,07 | 19,24 | 27,20 | 19,86 | 0,369 | 31,33 | 16,70 | 32,08 | 21,46 | 0,919 | 32,10 | 4,08 | 27,76 | 9,47 | 0,328 |
| RG score, scenario no. 4 | 27,00 | 8,99 | 33,36 | 7,09 | 0,099 | 27,17 | 9,78 | 31,10 | 6,47 | 0,262 | 28,54 | 8,98 | 28,44 | 9,10 | 0,977 | 32,02 | 7,00 | 29,48 | 8,61 | 0,541 |
| RG score, scenario no. 5 | 12,52 | 7,44 | 18,57 | 2,76 | **0,046** | 13,87 | 6,74 | 14,05 | 8,15 | 0,951 | 13,37 | 6,95 | 14,30 | 7,37 | 0,732 | 12,00 | 8,45 | 14,32 | 6,93 | 0,514 |
| RG score, all scenario | 132,38 | 44,26 | 159,74 | 31,41 | 0,141 | 139,99 | 41,68 | 136,31 | 47,08 | 0,828 | 136,14 | 41,77 | 140,52 | 44,53 | 0,789 | 134,72 | 43,58 | 139,58 | 43,47 | 0,821 |

* Anova test; ** NCRS scale: Scores range from 0 to 75. Scenario no. 1: scores range from 0 to 58. Scenario no. 2: scores range from 0 to 51. Scenario no. 3: scores range from 0 to 38. Scenario no. 4: scores range from 0 to 41. Scenario no. 5: scores range from 0 to 20. All scenarios: scores range from 0 to 208

**Table S4.** Correlation between NCRS scale and the game scores by age, gender, workplace setting, and number of matches played.

|  | Gender | | | | Nurse type | | | | Age classes | | | | Number of matches by scenario | | | |
| --- | --- | --- | --- | --- | --- | --- | --- | --- | --- | --- | --- | --- | --- | --- | --- | --- |
|  | Women | | Men | | Primary care nurses | | Other nurses | | <44 years | | ≥45 years | | One | | More than one | |
|  | Coeff. | p-value* | Coeff. | p-value* | Coeff. | p-value* | Coeff. | p-value* | Coeff. | p-value* | Coeff. | p-value* | Coeff. | p-value* | Coeff. | p-value* |
| RG score, scenario no. 1 | 0,021 | 0,930 | -0,164 | 0,650 | -0,215 | 0,503 | 0,038 | 0,881 | 0,022 | 0,921 | -0,324 | 0,478 | 0,400 | 0,505 | 0,025 | 0,905 |
| RG score, scenario no. 2 | 0,113 | 0,635 | -0,043 | 0,907 | -0,109 | 0,736 | 0,106 | 0,676 | 0,097 | 0,659 | 0,162 | 0,728 | 0,900 | **0,037** | -0,054 | 0,798 |
| RG score, scenario no. 3 | 0,100 | 0,676 | -0,317 | 0,374 | -0,554 | 0,061 | 0,442 | 0,066 | 0,031 | 0,888 | -0,180 | 0,699 | 0,667 | 0,219 | -0,204 | 0,329 |
| RG score, scenario no. 4 | -0,030 | 0,900 | -0,091 | 0,802 | -0,432 | 0,160 | -0,025 | 0,922 | -0,137 | 0,533 | 0,486 | 0,268 | -0,200 | 0,747 | -0,038 | 0,857 |
| RG score, scenario no. 5 | 0,020 | 0,934 | -0,219 | 0,543 | -0,109 | 0,735 | -0,355 | 0,149 | -0,103 | 0,640 | 0,109 | 0,816 | 0,205 | 0,741 | -0,087 | 0,679 |
| RG score, all scenarios | 0,050 | 0,835 | -0,231 | 0,521 | -0,284 | 0,371 | 0,002 | 0,993 | -0,061 | 0,781 | 0,018 | 0,969 | 0,400 | 0,505 | -0,001 | 0,994 |

* Spearman's Rank-Order Correlation
